# Tailoring Garlic Bioactive Profiles Through Green Extraction: Comparative Optimization of Microwave- and Ultrasound-Assisted Technologies

**DOI:** 10.3390/molecules31203629

**Published:** 2026-10-12

**Authors:** Marko Krstić, Nemanja Teslić, Filip Andrić, Perica Bošković, Branimir Pavlić

**Affiliations:** 1 Faculty of Chemistry, University of Belgrade, Studenski trg 16, 11158 Belgrade, Serbia; mkrstic109@gmail.com (M.K.); andric@chem.bg.ac.rs (F.A.); 2 Institute of Food Technology, University of Novi Sad, Bulevar cara Lazara 1, 21000 Novi Sad, Serbia; nemanja.teslic@fins.uns.ac.rs; 3 Department of Chemistry, Faculty of Science, University of Split, Ruđera Boškovića 33, 21000 Split, Croatia; 4 Faculty of Technology, University of Novi Sad, Bulevar cara Lazara 1, 21000 Novi Sad, Serbia; bpavlic@uns.ac.rs

**Keywords:** *Allium* species, phytochemicals, organosulfur compounds, allicin, phenolic compounds, antioxidant capacity, extraction selectivity, functional food ingredients

## Abstract

Garlic (*Allium sativum* L.) contains a wide range of bioactive compounds, including phenolic compounds, flavonoids, and organosulfur compounds. Efficient recovery of phenolic and organosulfur compounds from garlic requires extraction technologies capable of selectively preserving chemically distinct bioactive constituents. This study comparatively optimized microwave-assisted extraction (MAE) and ultrasound-assisted extraction (UAE) to elucidate their selectivity toward different classes of garlic bioactive compounds using response surface methodology (RSM) based on a central composite design. The effects of various extraction parameters on total phenolic content (TPC), total flavonoid content (TFC), antioxidant activity (DPPH, FRAP, and ABTS), and allicin content were investigated. Model performance varied by response: several equations supported prediction within the studied ranges, whereas the MAE TFC and UAE DPPH and ABTS models showed limited predictive ability and significant lack of fit despite the high predicted R^2^. At the selected conditions, the measured MAE extract contained 58.29 mg allicin/100 g DW after 7 min, whereas the measured UAE extract had TPC and TFC values of 358.7 g GAE/100 g DW and 117.8 g CE/100 g DW, respectively. These observations indicate that the selected conditions produced different bioactive profiles, but do not establish a statistically robust optimum for every response included in the desirability calculation.

## 1. Introduction

Garlic (*Allium sativum* L.) is one of the most studied plant species due to its high nutritional value and rich phytochemical composition. Its biological activity is associated with the presence of different groups of bioactive compounds, including phenolic compounds, flavonoids, and organosulfur compounds, among which allicin occupies a special place. These compounds contribute to the antioxidant, antimicrobial, and other functional properties of garlic [1,2]. Therefore, garlic extracts are increasingly being investigated as potential ingredients for functional foods, nutraceuticals, and natural products [3,4].

The recovery and preservation of these bioactive constituents during extraction are critical for obtaining garlic extracts with enhanced functional potential [5,6]. The efficiency of extracting target phytochemicals from plant matrices largely depends on the applied extraction method and process parameters, such as solvent composition, extraction time, temperature, liquid-solid ratio, and energy input [7,8]. Conventional extraction methods, such as maceration and Soxhlet extraction, often require long processing times and higher solvent consumption, and can degrade thermolabile compounds, thereby limiting their application in the development of sustainable extraction processes [9]. Therefore, in recent years, special attention has been directed towards the development of green extraction technologies that enable more efficient recovery of bioactive components with reduced energy and solvent consumption [10,11]. Various advanced extraction techniques have been investigated within this framework, including pressurized liquid extraction (PLE) and subcritical water extraction (SWE). These approaches have demonstrated high efficiency in recovering bioactive compounds from garlic, particularly phenolics and organosulfur constituents, while reducing organic solvent consumption [12].

Among modern green extraction technologies, microwave-assisted extraction (MAE) and ultrasound-assisted extraction (UAE) stand out due to their ability to enhance mass transfer, shorten extraction time, and improve the recovery of target compounds [13]. Although both techniques are classified as green extraction technologies, they rely on fundamentally different mechanisms of cell disruption and mass transfer, which may differently affect the recovery of thermolabile and chemically diverse garlic constituents. MAE is based on rapid volumetric heating of the extraction system due to dielectric polarization, which can lead to an increase in internal pressure within plant cells and facilitated release of intracellular components [14]. On the other hand, UAE acts primarily through the phenomenon of acoustic cavitation, whereby the formation and collapse of bubbles leads to mechanical disruption of the cellular structure, increasing tissue permeability and improving the diffusion of bioactive compounds into the extraction medium [15].

Despite the considerable potential of MAE and UAE, the extraction of garlic bioactive compounds remains challenging because of the chemical diversity of the target constituents. Phenolic compounds, flavonoids, and organosulfur compounds differ markedly in their polarity, stability, and extraction behavior [16,17], making their simultaneous recovery under a single set of operating conditions difficult. In particular, allicin is highly reactive and susceptible to degradation during processing, whereas phenolic compounds generally require extraction systems with appropriate solvent polarity to achieve high recovery. Water-ethanol systems are widely used for the extraction of phenolic and other bioactive compounds due to their tunable polarity, low toxicity, and suitability for application in the food industry [7,18]. Consequently, extraction conditions must be carefully selected not only to maximize extraction yield but also to control the selectivity of the process toward specific groups of bioactive compounds.

The complexity of extraction systems makes the simultaneous optimization of multiple quality parameters challenging. Multicriteria approaches, such as the desirability function, provide an effective tool for identifying compromise optimal conditions and selecting the most suitable process parameters [19]. Response Surface Methodology (RSM) is an effective statistical approach for optimizing complex extraction processes, as it allows for the assessment of the influence of individual factors, their interactions, and the definition of optimal process conditions with a reduced number of experimental tests. This approach has been successfully applied to optimize the extraction of various bioactive compounds from plant materials, including phenolic components and antioxidants [20,21]. In our previous study, pressurized liquid extraction (PLE) and subcritical water extraction (SWE) were successfully applied for the recovery of garlic bioactive compounds, demonstrating the importance of extraction conditions in modulating the yield of phenolics, organosulfur compounds, and antioxidant activity [12]. Although these high-pressure extraction techniques provide excellent extraction efficiency, they require specialized equipment, more initial investment on an industrial level, and elevated operating conditions, highlighting the need to investigate more accessible green extraction technologies. Therefore, MAE and UAE, which operate under milder conditions while maintaining high extraction efficiency, represent promising alternatives for garlic valorization. However, comparative studies evaluating MAE and UAE for the recovery of different groups of garlic phytochemicals while simultaneously optimizing multiple extract quality parameters remain limited.

Although MAE and UAE have individually been investigated for the extraction of phytochemicals [13,14,15], studies directly comparing these technologies under statistically optimized conditions and simultaneously considering phenolic compounds, flavonoids, antioxidant capacity, and allicin recovery remain scarce. Therefore, optimizing extraction conditions is essential to maximize the recovery of this compound while preserving other valuable garlic constituents.

The aim of this study was to comparatively optimize MAE and UAE for the selective recovery of garlic bioactive compounds using response surface methodology (RSM) coupled with a central composite design (CCD). The influence of key process variables on total phenolic content (TPC), total flavonoid content (TFC), antioxidant activity (DPPH, FRAP, and ABTS), and allicin content was systematically evaluated, and the developed models were used to identify optimal extraction conditions. Furthermore, the optimized MAE and UAE processes were comparatively assessed to determine their suitability for producing garlic extracts with tailored chemical profiles, thereby providing a scientific basis for selecting green extraction technologies according to the desired composition and intended application of the final extract.

## 2. Results and Discussion

### 2.1. Microwave-Assisted Extraction (MAE)

#### 2.1.1. Experimental Results

The experimental results obtained according to the central composite design (CCD) are summarized in Table 1. The developed models evaluated the effects of four independent process variables, namely ethanol concentration, extraction time, liquid-to-solid ratio, and microwave radiation power, on the selected response variables, including total phenolic content (TPC), total flavonoid content (TFC), antioxidant activity determined by DPPH, FRAP, and ABTS assays, and allicin content.

Considerable variability among the experimental runs was observed for all response variables, demonstrating the strong dependence of microwave-assisted extraction (MAE) performance on operating conditions. Such variability is expected because MAE is governed by the combined effects of solvent polarity, microwave energy absorption, internal heating, and mass transfer, all of which influence the release and stability of chemically diverse garlic constituents [22]. Total phenolic content (TPC) varied more than threefold, ranging from 96.88 to 325.11 g GAE/100 g DW, while total flavonoid content (TFC) ranged from 14.36 to 44.34 g CE/100 g DW. This wide variation indicates that MAE conditions substantially influenced the extraction efficiency of phenolic constituents [9]. Similar trends were observed for antioxidant activity, with DPPH, FRAP, and ABTS values varying considerably across the experimental domain. Since antioxidant capacity reflects the combined contribution of multiple reducing and radical-scavenging compounds rather than a single phytochemical, these differences suggest that extraction conditions altered not only the quantity but also the composition of the recovered bioactive constituents [10].

Allicin content ranged from 13.13 to 61.20 mg/100 g, which indicates a significant variability of this response depending on the applied extraction conditions. This pronounced variability reflects the well-documented chemical instability of allicin, which is highly susceptible to degradation during thermal processing and prolonged extraction [1]. This highlights the importance of process optimization to obtain garlic extracts with a targeted bioactive profile.

#### 2.1.2. Model Adequacy

Adequacy of the developed regression models was evaluated based on analysis of variance (ANOVA) and statistical parameters, including the coefficient of determination (R^2^), adjusted R^2^, predicted R^2^, and lack-of-fit test (Table 2). The developed quadratic models showed different levels of adequacy depending on the investigated response, with R^2^ values ranging from 0.8326 to 0.9765. The MAE models differed in their suitability for prediction. TPC, DPPH, FRAP, and ABTS showed no significant lack of fit, whereas the TFC model had a negative predicted R^2^ (−0.0270) and significant lack of fit (*p* = 0.0195). The allicin model had a relatively high predicted R^2^ (0.8775), but its significant lack of fit (*p* = 0.0050) indicates that its predictions also require caution. Accordingly, the TFC equation should not be treated as a reliable predictive model, and the allicin equation cannot be described as fully adequate on the basis of R^2^ alone.

The TFC model exhibited lower predictive capability compared with the other responses, as indicated by its negative predicted R^2^ value and significant lack-of-fit (*p* = 0.0195). This suggests that the quadratic model had limited ability to predict TFC values within the investigated experimental range, although the model remained statistically significant. Therefore, the TFC response was interpreted with caution when assessing the effects of individual process variables and defining optimal extraction conditions. The allicin model exhibited a high coefficient of determination (R^2^ = 0.9765) and excellent predictive performance (predicted R^2^ = 0.8775). However, the significant lack-of-fit (*p* = 0.0050) indicates that some unexplained variability remained, and therefore predictions obtained from this model should be interpreted within the investigated experimental range.

Overall, these results support the applicability of the RSM approach for evaluating the influence of MAE operating conditions on the recovery of garlic bioactive compounds. Similar RSM-based optimization approaches have been successfully applied to optimize the extraction of valuable phytochemicals from different plant matrices [23,24,25].

#### 2.1.3. Effect of MAE Variables on Phenolic Compounds, Antioxidant Activity and Allicin

**Figure 1:**
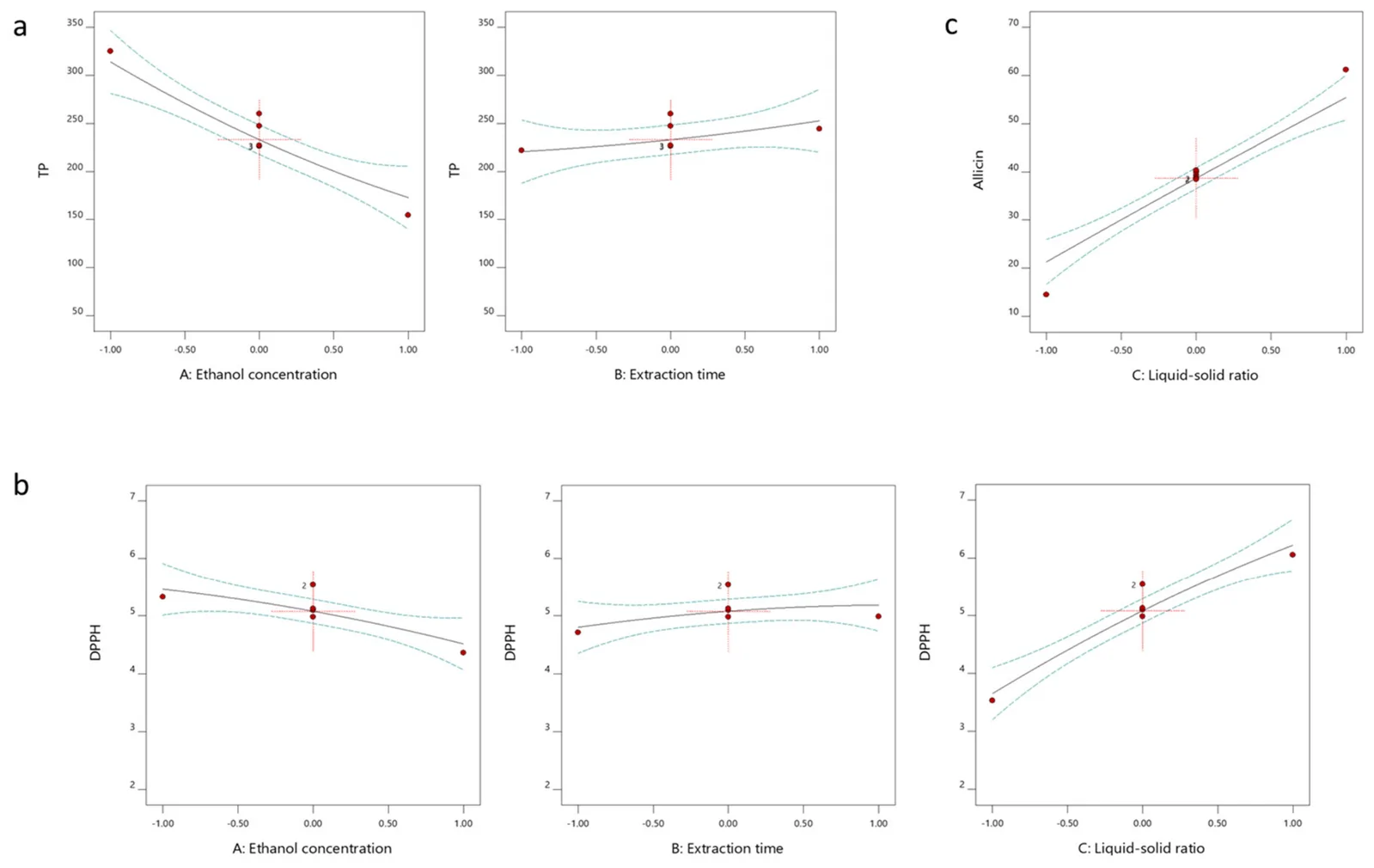
Perturbation diagrams of the significant process factors affecting (**a**) TPC (ethanol concentration and extraction time), (**b**) DPPH (ethanol concentration, extraction time and liquid-solid ratio), and (**c**) allicin content (liquid-solid ratio) during the MAE process.

Ethanol concentration was identified as the dominant factor governing the recovery of phenolic compounds during MAE, as confirmed by the ANOVA results and perturbation plots (Figure 1). The perturbation plots indicated that TPC was favored by lower ethanol concentrations, suggesting that a higher proportion of water in the extraction solvent enhanced the recovery of phenolic constituents. This behavior can be attributed to the higher affinity of polar phenolic compounds for aqueous ethanol mixtures and the improved swelling of plant tissues, which facilitates the diffusion of intracellular compounds into the extraction medium [7,18]. The higher extraction efficiency observed at lower ethanol concentrations can be additionally attributed to the ability of water to enhance microwave energy absorption owing to its high dielectric constant. Increased dielectric heating promotes rapid internal heating of plant tissues, facilitating cell rupture and improving solvent penetration and mass transfer [14]. Extraction time also had a significant positive effect on TPC, indicating that prolonged microwave treatment enhanced mass transfer and promoted the release of phenolic compounds from the garlic matrix. In contrast, TFC was significantly affected by ethanol concentration, extraction time, and liquid-to-solid ratio, suggesting that flavonoid recovery depended on the combined influence of solvent polarity and extraction accessibility. Microwave power did not exert a statistically significant individual effect within the investigated experimental domain. This finding suggests that once sufficient microwave energy was supplied to initiate efficient heating, solvent composition became the principal factor controlling extraction efficiency which is in accordance with our previous studies [26].

Similar response trends were observed in antioxidant responses. Ethanol concentration was the dominant factor for DPPH, FRAP and ABTS values, while extraction time and liquid-solid ratio had an additional significant contribution to certain responses, especially DPPH and ABTS. Microwave power did not show a statistically significant individual effect within the tested experimental range. This suggests that variations in microwave power within the investigated range were not the dominant factor affecting extraction efficiency, whereas the properties of the extraction medium played a more important role in determining the recovery of antioxidant compounds. These results are in line with previous research on the extraction of bioactive components from *Allium* species, in which solvent composition was identified as one of the key factors determining the recovery of phenolic compounds and antioxidant capacity of plant extracts [27,28]. The similar response trends observed for DPPH, FRAP, and ABTS suggest that MAE conditions affected the overall antioxidant profile of garlic extracts, although differences among individual assays reflect their specific reaction mechanisms and selectivity toward different antioxidant compounds [29].

In contrast to phenolic compounds, allicin exhibited markedly greater sensitivity to extraction conditions, reflecting its highly reactive and chemically unstable nature. This is expected considering the specific chemical nature of organosulfur compounds from garlic, whose extraction depends on the availability of solvents, mass transfer and preservation of thermolabile components [30,31,32]. The liquid-solid ratio was the only statistically significant factor, which indicates that the availability of the extraction medium played a more important role than the other examined parameters. This behavior may be related to the specific mechanism of allicin formation, which occurs through the enzymatic conversion of alliin into allicin after the disruption of garlic cellular structures [33,34]. These findings indicate that successful recovery of allicin requires a careful balance between efficient tissue disruption, rapid solvent extraction, and preservation of this highly labile organosulfur compound. Consequently, MAE conditions should be selected not only to maximize extraction yield but also to preserve the integrity of sulfur-containing bioactives.

#### 2.1.4. Multi-Response Optimization

The original MAE desirability calculation included TPC, TFC, DPPH, FRAP, ABTS, and allicin. TFC was retained to represent flavonoid recovery, and allicin was retained as a central target of the study. However, the poor predictive performance of the TFC model and the significant lack of fit of the allicin model limit confidence in their contributions to the calculated solution. The selected conditions and overall desirability of 0.967 (Table 3) describe an exploratory compromise under the specified response goals and limits; a high desirability value does not establish predictive adequacy of the underlying models. Although the TFC model showed limited predictive ability, this response was retained due to its relevance for evaluating the overall bioactive profile of garlic extracts [26]. One extraction was performed at each selected optimum. The values reported as mean ± standard deviation in Table 3 and Table 6 represent the analytical replication procedure and do not represent independent extraction experiments. The comparison between predicted and observed values therefore provides a descriptive check at the selected conditions; it does not constitute an independent statistical validation of model predictions. This limitation has been taken into account when interpreting the optimized conditions.

The optimal solution defined by the model included lower ethanol concentration, shorter extraction time, higher liquid-to-solid ratio, and moderate microwave power (Table 3). The high desirability value (0.967) indicates that the developed models enabled the identification of suitable compromise conditions for simultaneous optimization of multiple extraction responses. The selected conditions represent a balance between efficient solvent accessibility, enhanced mass transfer, and preservation of sensitive garlic bioactive compounds.

### 2.2. Ultrasound-Assisted Extraction (UAE)

#### 2.2.1. Experimental Results

The experimental results obtained using the CCD design for the optimization of UAE are shown in Table 4. Models were developed for three process variables: extraction temperature, extraction time and ethanol concentration, while TPC, TFC, antioxidant activity determined by DPPH, FRAP and ABTS methods, as well as allicin content, were analyzed as responses.

The TPC values obtained by the UAE process ranged from 83.30 to 370.03 g GAE/100 g dry weight, whereas TFC ranged from 19.40 to 131.90 g CE/100 g dry weight. The pronounced variability observed for both responses indicates that UAE conditions substantially affected the extraction efficiency of phenolic constituents. Ultrasound-assisted extraction enhances the recovery of these compounds primarily through acoustic cavitation, which disrupts plant tissues, improves solvent penetration, and accelerates the diffusion of intracellular metabolites into the extraction medium [15].

The antioxidant potential of the extracts varied significantly depending on the experimental conditions. DPPH activity ranged from 0.93 to 2.56 mM TE/g, while FRAP and ABTS values ranged from 5.26 to 16.97 mM Fe^2+^/g and from 5.90 to 7.78 mM TE/g, respectively. The differences between individual antioxidant responses reflect the different selectivity of the applied assays toward specific groups of bioactive compounds present in the extracts.

The content of allicin ranged from 24.19 to 35.02 mg/100 g, exhibiting considerably lower variability than phenolic and flavonoid compounds. This observation suggests that, within the investigated experimental domain, UAE parameters exerted a less pronounced influence on organosulfur recovery than on phenolic extraction. Applied ultrasonic conditions may have been sufficient to promote efficient tissue disruption while minimizing extensive changes in allicin recovery. Considering the high chemical reactivity and limited stability of allicin, the relatively narrow response range further suggests that ultrasound processing within the investigated conditions preserved this organosulfur compound without inducing substantial degradation.

#### 2.2.2. Model Adequacy

All five UAE equations were statistically significant (*p* < 0.05), but they did not all provide adequate predictions (Table 5). The TPC, TFC, and FRAP models showed stronger predictive performance. The DPPH model had a predicted R^2^ of 0.1854 and significant lack of fit (*p* = 0.0080), while the ABTS model had a predicted R^2^ of −0.8015 and significant lack of fit (*p* = 0.0007). Thus, model significance alone does not support using DPPH and ABTS to make reliable predictions at untested conditions within the investigated domain.

The models developed for TPC, TFC, and FRAP exhibited satisfactory predictive performance, demonstrating good agreement between experimental and predicted values. In contrast, the DPPH model had a low predicted R^2^ (0.1854) and significant lack of fit (*p* = 0.0080), while the ABTS model had a negative predicted R^2^ (−0.8015) and significant lack of fit (*p* = 0.0007). Therefore, these models (DPPH and ABTS) were primarily used to describe response trends within the investigated experimental domain rather than for reliable extrapolation beyond the tested conditions. Significant lack of fit indicates that the quadratic equations did not fully capture these responses, and their low or negative predicted R^2^ values limit their usefulness for selecting conditions that maximize antioxidant activity. Accordingly, conclusions about the effects of UAE conditions and the resulting optimum are based primarily on the better-supported TPC, TFC, and FRAP models.

Ethanol concentration emerged as the dominant process variable for most responses, highlighting the central role of solvent polarity in UAE, according to ANOVA results. The polarity of aqueous ethanol mixtures governs not only the solubility of phenolic compounds but also cavitation efficiency, solvent penetration into plant tissues, and the diffusion of released metabolites into the extraction medium [35]. Extraction temperature showed no significant independent effect within the investigated range, suggesting that the mild temperatures employed during UAE were sufficient to maintain efficient mass transfer without substantially altering extraction performance, while extraction time had an additional influence on certain parameters. The obtained results confirm the importance of solvent selection in the UAE process, where the polarity of the extraction system directly affects the ability to extract phenolic and flavonoid components. The application of the RSM approach proved suitable for describing the UAE process, which is in line with previous research confirming the successful application of this methodology for the optimization of UAE of plant bioactive compounds [35,36].

#### 2.2.3. Effect of UAE Variables on Phenolic Compounds, Antioxidant Activity and Allicin

**Figure 2:**
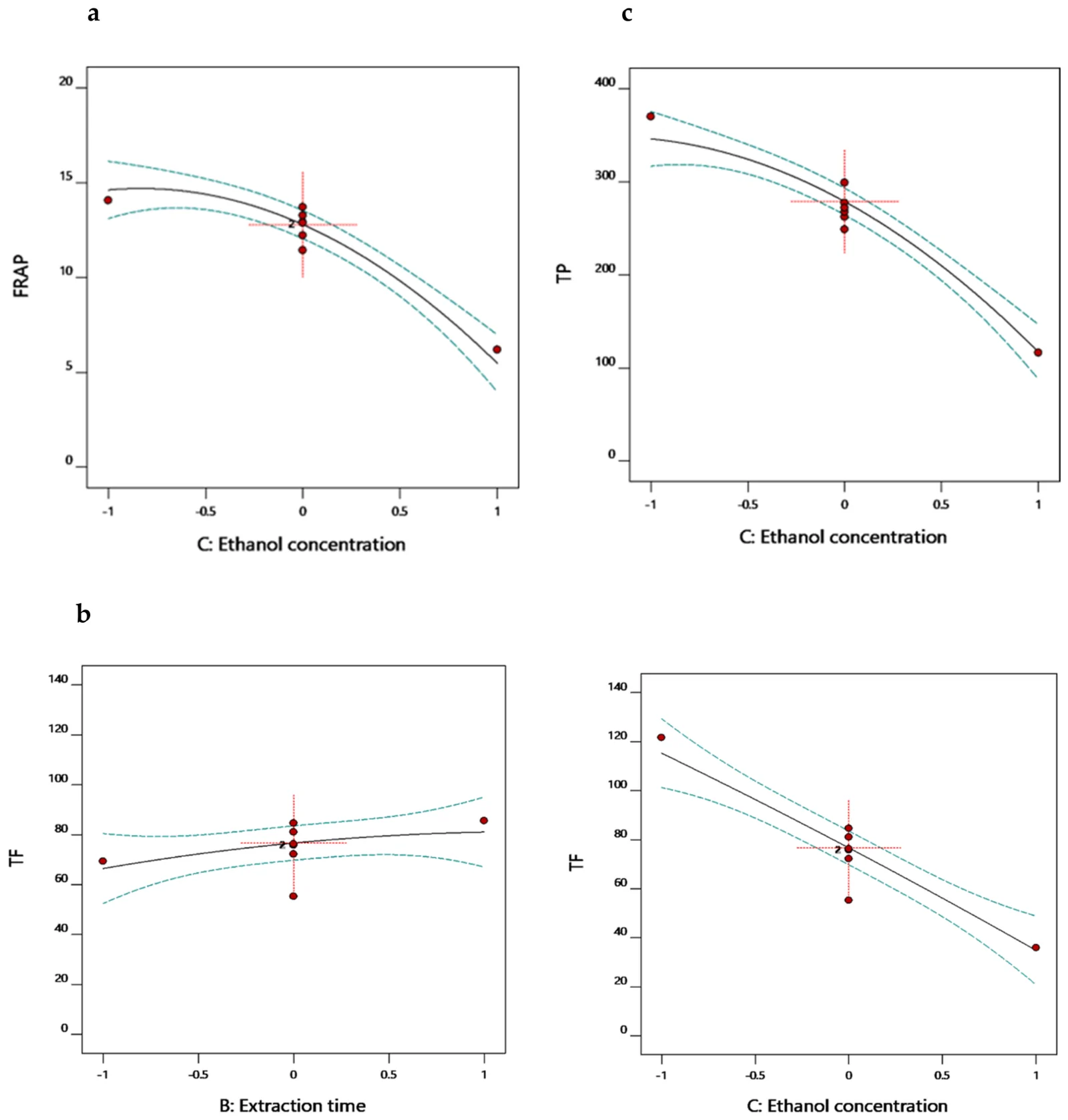
Perturbation diagrams of the significant process factors affecting (**a**) FRAP (ethanol concentration) during the UAE process TPC (ethanol con-centration), (**b**) TFC (ethanol concentration and extraction time), and (**c**) TPC (ethanol concentration).

The influence of individual process parameters on the investigated responses was analyzed using perturbation diagrams (Figure 2).

Ethanol concentration was the most significant process factor that determined the efficiency of extraction of phenolic and flavonoid compounds. Increasing ethanol concentration within the investigated range resulted in lower TPC and TFC values, indicating the importance of the appropriate polarity of the extraction system for efficient separation of these compounds. Extraction time had an additional significant contribution to individual responses, while extraction temperature did not show a statistically significant individual effect within the applied experimental range. The absence of a significant effect of temperature may indicate that the contribution of acoustic cavitation had a more significant role than the thermal effect within the investigated range, while an additional increase in temperature did not significantly improve mass transfer [35].

In antioxidant responses, different patterns of dependence on process factors were observed. Ethanol concentration was the dominant factor for most analyzed antioxidant parameters, while extraction time had an additional significant effect on ABTS responses. Extending the extraction time had a positive effect on certain antioxidant responses, especially FRAP and ABTS, while the extraction temperature did not show a significant individual effect within the tested experimental range. Prolonged ultrasound treatment may enhance acoustic cavitation and facilitate the release of intracellular bioactive compounds through mechanical disruption and increased matrix permeability [15,35,37]. The absence of a significant effect of temperature indicates that within the examined experimental range, the thermal contribution was not a limiting factor of the process. The differences in the behavior of individual antioxidant responses can be related to the different selectivity of the applied determination methods and different reaction mechanisms with the bioactive components present in the extracts [29].

The lower variability of allicin content in response to UAE parameters may indicate that the investigated range of ultrasonic conditions was not sufficient to significantly affect its release or degradation. Therefore, allicin content was not included as an optimization criterion of the UAE process. This behavior is consistent with the claims that the response of organosulfur compounds to UAE depends on their chemical nature, stability, and mechanism of release from the plant matrix [35].

These findings indicate that UAE was more effective for modulation of phenolic and antioxidant profiles than for controlling allicin recovery under the investigated conditions.

#### 2.2.4. Multi-Response Optimization

The original UAE desirability calculation included TPC, TFC, DPPH, FRAP, and ABTS. DPPH and ABTS were retained to represent distinct antioxidant assays, although their weak predictive performance and significant lack of fit limit the reliability of their contributions. Consequently, the conditions presented in Table 6 are an exploratory multi-response compromise within the investigated ranges, supported more strongly by the TPC, TFC, and FRAP models. They should not be interpreted as reliably maximizing DPPH or ABTS.

The single extraction performed at the selected UAE conditions yielded values numerically close to the model predictions, particularly for TPC and TFC (Table 6). This is a descriptive experimental check at one set of conditions. The analytical replicates do not provide independent evidence of extraction reproducibility or establish the predictive accuracy of the models. The significant lack of fit and weak predicted R^2^ values for DPPH and ABTS therefore remain important limitations.

### 2.3. Comparative Evaluation of MAE and UAE Extraction Performance

MAE and UAE were optimized separately using different process variables and experimental ranges. MAE was investigated over extraction times of 1–7 min with liquid-to-solid ratio and microwave power as additional factors, whereas UAE was investigated over 20–60 min with extraction temperature as an additional factor and a fixed liquid-to-solid ratio. The results below therefore compare the extracts obtained at each technology’s selected operating conditions.

A comparative analysis of the MAE and UAE processes showed that both technologies represent effective approaches for the extraction of bioactive compounds from garlic, but that they differ in their selectivity toward individual groups of bioactive compounds and process requirements. The comparison was made based on the separately selected conditions checked by one extraction per method, which provided a more reliable assessment of the actual performance of both extraction technologies. The differences between the methods can be related to different mechanisms of energy transfer: MAE enables rapid volumetric heating of the system due to dielectric polarization [22,38], while UAE enhances extraction primarily through acoustic cavitation, which contributes to the disruption of cell structure and intensification of mass transfer [9,39]. 

At the selected conditions, the UAE extract had higher measured TPC and TFC values (358.7 g GAE/100 g DW and 117.8 g CE/100 g DW) than the MAE extract (321.8 g GAE/100 g DW and 40.21 g CE/100 g DW; Table 3 and Table 6). These numerical differences describe one extraction at each selected setting. Because the methods were evaluated under different operating conditions and the selected settings were not independently replicated, they do not establish that UAE is generally more efficient at recovering phenolic or flavonoid compounds.

The TPC values obtained were comparable to the results previously published using the same garlic genotype (Ranco) and comparable experimental material, where, using pressure extraction (PLE), the content of phenolic compounds ranged from 92.36 to 391.45 g GAE/100 g [12]. The maximum value achieved by the PLE process was close to the values obtained by the UAE process in this work, but with the application of high pressure and temperature of 110 °C. This indicates that UAE may represent an effective alternative for the extraction of phenolic compounds under milder process conditions.

At the selected conditions, measured allicin content was 58.29 mg/100 g for MAE and 27.52 mg/100 g for UAE (Table 3 and Table 6). This numerical difference may reflect the combined effects of extraction time, solvent conditions, and the distinct energy-transfer mechanisms, but their individual contributions cannot be separated by the present comparison. The previously reported PLE values for the same garlic genotype (16.03–24.04 mg/100 g) [12] provide context; they do not support a claim of a statistically significant difference between studies.

The comparison of antioxidant activity showed that the choice of extraction technology does not simply determine the total antioxidant potential, but affects the ratio of different bioactive components in the extract. Although UAE provided a higher content of phenolic and flavonoid compounds, MAE extracts showed a high antioxidant potential, which can be related to a higher content of allicin and other organosulfur compounds. These results confirm that the antioxidant activity is the result of the joint contribution of different groups of compounds and that the interpretation of one chemical parameter is not sufficient to evaluate the functional potential of the extract [29]. The higher allicin content obtained by MAE further confirms the suitability of the applied conditions for the recovery of this organosulfur compound.

The selected MAE extraction time was 7 min, compared with 52.8 min for the selected UAE treatment. MAE therefore required a shorter extraction step at these particular settings. This comparison does not account for preparation, cooling, energy use, or other aspects of overall process efficiency. Because the methods were optimized over different experimental domains, the observed time difference should not be interpreted as a general measure of their relative performance. 

The single-extraction results at the selected conditions suggest different extract profiles: the MAE extract had higher measured allicin content, whereas the UAE extract had higher measured TPC and TFC. These observations may inform the selection of conditions for further testing when a particular bioactive profile is desired. Replicated experiments and a comparative design would be needed to establish reproducible between-method differences or determine whether either technology performs better under comparable conditions. The differences observed between MAE and UAE can be related to the distinct mechanisms by which energy is transferred to the extraction system. In MAE, microwave energy is converted into heat through dielectric interactions with the solvent and plant matrix, enabling rapid and volumetric heating. The higher efficiency of microwave energy transfer may promote rapid disruption of cellular structures and accelerate solvent penetration and mass transfer, which is particularly relevant for short extraction times. In contrast, UAE relies primarily on acoustic cavitation, in which the formation and collapse of microscopic bubbles generates localized mechanical effects that can disrupt plant tissues, increase matrix permeability, and facilitate the release of intracellular compounds. Thus, although both technologies enhance mass transfer and improve extraction efficiency, their mechanisms of energy transfer differ substantially. The observed differences in the recovery of phenolic compounds, flavonoids, antioxidant responses, and allicin therefore indicate that MAE and UAE should be considered complementary rather than interchangeable extraction approaches, with the choice of technology depending on the targeted bioactive profile and the desired processing conditions.

The higher allicin recovery observed under MAE may be related to the rapid energy transfer and short extraction time, which may facilitate the release of allicin already formed during the preceding sample preparation while limiting the duration of exposure to extraction conditions that could promote its degradation. In the present study, the garlic material was allowed to stand for 30 min after cutting before drying, providing time for enzymatic conversion of alliin to allicin prior to the extraction step. Therefore, the contribution of residual alliinase activity during MAE cannot be established from the present experiments and should be considered speculative. The observed higher allicin content may instead reflect the combined effects of rapid matrix disruption, efficient mass transfer, and the short duration of microwave treatment. Nevertheless, because allicin is highly reactive and thermolabile, the possibility that microwave heating also affected its degradation pathways cannot be excluded and would require targeted experiments for confirmation. Comparison with the previously reported PLE results should likewise be interpreted cautiously, since the studies differed in extraction temperature, pressure, solvent conditions, sample preparation, and extraction time [12].

## 3. Materials and Methods

### 3.1. Chemicals

The following reagents were used in the research: 1,1-diphenyl-2-picrylhydrazyl hydrate (DPPH), 2,2′-azino-bis(3-ethylbenzothiazoline-6-sulfonic acid) diammonium salt (ABTS•^+^), Trolox (6-hydroxy-2,5,7,8-tetramethylchroman-2-carboxylic acid), Folin–Ciocalteu reagent and (+)-catechin, purchased from Sigma-Aldrich GmbH (Steinheim, Germany). Gallic acid was purchased from Sigma (St. Louis, MO, USA), while potassium persulfate (99% purity) was provided by Acros Organics (Geel, Belgium). All other chemicals and reagents were of analytical grade and were used without further purification.

### 3.2. Plant Material

The research used garlic (*Allium sativum* L.), genotype Ranco, grown in the experimental field “Rimski Šančevi” of the Institute of Agriculture and Vegetables in Novi Sad. The plant material was produced in controlled field conditions using the Institute’s standard agrotechnical measures. After harvesting, the bulbs were visually inspected, and only healthy, fully developed samples without visible symptoms of disease, mechanical damage, and physiological changes were selected for further testing.

### 3.3. Sample Preparation

Fresh heads of garlic (*Allium sativum* L.) were manually peeled, cut into smaller pieces and left to stand for 30 min at room temperature to allow enzymatic conversion of organosulfur compound precursors. Approximately 200 g of prepared plant material was dried in a laboratory vacuum dryer according to the previously optimized procedure described by Vakula et al. [40]. During the process, the heating surface temperature, chamber pressure and sample mass were automatically monitored and recorded using a programmable logic controller (PLC). The mass of the sample was determined at intervals of 10 min, and drying was continued until a constant mass was reached, defined as three consecutive measurements without a significant change in mass. Based on previously optimized conditions, drying was carried out at a heating surface temperature of 78 °C, a pressure of 30 mbar, and a total duration of 11 h. After completion of the process, the moisture content of the dried samples was 4.79%. 

The entire dried material was subsequently milled and combined into a single homogeneous batch of powdered garlic. The prepared material was stored together in a closed container protected from moisture and light. This same homogeneous batch of dried and milled garlic was used as the starting material for all extraction experiments in the present study, including all MAE and UAE experimental runs. Thus, the same plant material, genotype, cultivation conditions, harvest season, post-harvest handling, drying and milling procedure were used throughout the experimental design, minimizing variability unrelated to the extraction technology and enabling a more consistent comparison of MAE and UAE.

The same garlic genotype, cultivation site, post-harvest handling, storage conditions, and sample preparation procedure were also used in our previous study [12], in which the corresponding garlic material was subjected to pressure-assisted extraction. The use of the same prepared and homogenized garlic material was intended to minimize variability associated with plant material and sample preparation and to facilitate meaningful comparison between the present study and our previous work.

### 3.4. Microwave-Assisted Extraction (MAE)

MAE was applied to isolate bioactive compounds from dried garlic (*Allium sativum* L., genotype Ranco) using an aqueous ethanol solution as an extraction solvent. The experiments were conducted in a laboratory microwave oven (MM817ASM, Bosch, Gerlingen, Germany). The influence of four independent process variables on extraction efficiency was investigated using RSM. Ethanol concentration (45–75%, *v*/*v*), extraction time (1–7 min), liquid/solid ratio (10–30 mL/g) and microwave power (400–800 W) were selected as independent variables. The experimental plan was based on a face-centered central composite design (CCD) with four independent variables and comprised 29 experimental runs. The design consisted of 16 factorial points, 8 axial points and 5 replicated center points, corresponding to a face-centered configuration with α = 1. The factor levels were coded as −1, 0 and +1, with the actual values corresponding to the low, center and high levels presented in Table 7. This design structure was selected to enable estimation of linear, interaction and quadratic effects of the investigated MAE variables within the defined experimental domain. 

For each experiment, 50 mL of extraction solvent was prepared, while the mass of the dried sample was adjusted according to the selected ratio of liquid and solid matter. The sample and solvent were homogenized immediately before the start of the extraction, after which the appropriate microwave treatment regime defined by the experimental design was applied. In each experimental run, the sample was mixed with 50 mL of extraction solvent in 500 mL round glass flasks. Flasks were then placed in the MAE apparatus and extractions were performed at fixed frequency (2450 MHz). Flasks were always positioned at the same distance from the magnetron and no additional agitation was applied. Extractions were performed in open mode by fixing a condenser on the extraction flask. Temperature was not monitored nor controlled during the process and extractions were performed at the solvent’s boiling point. After extraction, crude extracts were immediately filtered through filter paper of 4–12 µm pore size (Schleicher & Schuell, Dassel, Germany) under vacuum. Extracts were collected into glass flasks and stored at 4 °C until further analysis.

### 3.5. Ultrasound-Assisted Extraction (UAE)

UAE was applied to isolate bioactive compounds from dried garlic (*Allium sativum* L., genotype Ranco) using an aqueous ethanol solution as an extraction solvent. The experiments were carried out in an ultrasonic water bath (EUP540A, Euinstruments, Paris, France) with a fixed frequency at 40 kHz. Process optimization was performed using RSM, where extraction temperature (30–50 °C), extraction time (20–60 min) and ethanol concentration (45–75%, *v*/*v*) were selected as independent process variables. The experimental plan was based on a face-centered central composite design (CCD) with three independent variables and a total of 20 experimental runs, comprising 8 factorial points, 6 axial points, and 6 replicated center points (α = 1). The factor levels were coded as −1, 0, and +1, with the corresponding actual values presented in Table 7. The investigated ranges were 30–50 °C for extraction temperature, 20–60 min for extraction time, and 45–75% (*v*/*v*) for ethanol concentration. These experimental ranges were selected based on preliminary experiments performed prior to the response surface optimization, with the aim of defining a suitable experimental domain for the UAE process. For each experiment, 2.5 g of dried powdered sample was weighed, to which 50 mL of extraction solvent was added in a 150 mL Erlenmeyer flask. During all experiments, the ultrasonic power was maintained at 70% of the ultrasonic power which corresponds to the 42 W/L effective power, while all other operating parameters remained constant. The working volume of the ultrasonic water bath was 6 L and ultrasonic power, temperature and extraction time were controlled from the panel of the instrument. In order to prevent evaporation of the extraction solvent, a condenser was added to the flask during extraction. Flasks were always positioned in the position of the ultrasonic bath in order to provide constant ultrasonic power. After extraction, extracts were filtered through filter paper, collected into glass vials, sealed and stored at 4 °C prior analysis.

For practical reasons related to instrument operation, the experimental runs were organized in groups according to operating temperature, allowing consecutive experiments at the same or similar temperature and minimizing repeated cooling and re-stabilization of the equipment.

### 3.6. General Procedure for Extract Handling

After the completion of the extraction procedures (MAE and UAE), the obtained extracts were immediately subjected to vacuum filtration through filter paper to remove undissolved particles. The filtrates were quantitatively transferred into volumetric flasks and supplemented with the appropriate extraction solvent to a final volume of 50 mL. The prepared extracts were stored at −20 °C until analyses. This storage condition was applied to minimize degradation of thermolabile compounds, particularly allicin, and to preserve the chemical profile of the extracts until analysis. Before analysis, the samples were thawed at room temperature and, according to the requirements of each analytical method, appropriately diluted so that their concentrations were within the linear range of the applied spectrophotometric and chromatographic methods. Spectrophotometric assays were selected for the determination of total phenolic content, total flavonoid content, and antioxidant activity, as they represent widely accepted, rapid, and reliable approaches for the characterization of plant extracts, while allicin was determined by chromatographic assay.

### 3.7. Total Phenolic Content (TPC)

The total content of phenolic compounds (TPC) in garlic extracts was determined using the Folin–Ciocalteu colorimetric method [26]. An aliquot of 0.1 mL of the appropriately diluted extract was mixed with 0.75 mL of diluted Folin–Ciocalteu reagent and allowed to react for 5 min at room temperature. After that, 0.75 mL of 6% (*w*/*v*) sodium carbonate solution was added, and the reaction mixture was incubated for 90 min at room temperature in the dark for complete color development. At the end of the incubation, the absorbance was measured at 750 nm using a UV–Vis spectrophotometer (6300 Spectrophotometer, Jenway, Stone, UK). A calibration curve was constructed using gallic acid as a reference standard, and the results were expressed as grams of gallic acid equivalents per 100 g of dry weight (g GAE/100 g DW).

### 3.8. Total Flavonoid Content (TFC)

Total flavonoid content (TFC) in garlic extracts was determined by a spectrophotometric colorimetric method with aluminum chloride [26]. Briefly, 0.5 mL of an appropriately diluted extract was mixed with 0.5 mL of a 2% (*w*/*v*) aluminum chloride solution prepared in methanol. The reaction mixture was incubated for 10 min at room temperature, after which the absorbance at 425 nm was measured using a UV–Vis spectrophotometer (6300 Spectrophotometer, Jenway, Stone, UK). A calibration curve was prepared using quercetin as a reference standard. The results are expressed as grams of quercetin equivalents per 100 g of dry weight (g CE/100 g DW).

### 3.9. DPPH Radical Scavenging Assay

The antioxidant activity of garlic extracts was determined using the DPPH test (2,2-diphenyl-1-picrylhydrazyl) according to the method described by Pavlić et al. [26]. A freshly prepared solution of DPPH in methanol was adjusted to an initial absorbance of 0.70 ± 0.02 at 517 nm and stored protected from light until use. For analysis, 0.1 mL of appropriately diluted extract was mixed with 2.9 mL of DPPH solution. The reaction mixture was incubated for 60 min at room temperature in the dark, after which the decrease in absorbance was measured at 517 nm using a UV–Vis spectrophotometer (6300 Spectrophotometer, Jenway, Stone, UK). Calibration curves were constructed using Trolox standard solutions in the concentration range 0–0.8 mM. Results are expressed as millimoles of Trolox equivalents per gram of dry weight (mM TE/g DW).

### 3.10. Ferric Reducing Antioxidant Power (FRAP) Assay

The antioxidant capacity of garlic extracts was determined using the FRAP method (Ferric Reducing Antioxidant Power) according to the procedure described by Pavlić et al. [26]. The method is based on the reduction of the complex iron (III)-2,4,6-tripyridyl-s-triazine (Fe^3+^–TPTZ) into the intensely colored complex iron (II)-TPTZ (Fe^2+^–TPTZ) in the presence of antioxidant compounds. The FRAP reagent was prepared immediately before analysis by mixing 300 mM acetate buffer (pH 3.6), 10 mM TPTZ solution prepared in 40 mM HCl, and 20 mM iron (III)-chloride (FeCl_3_) solution in a volume ratio of 10:1:1 (*v*/*v*/*v*). For the determination of antioxidant capacity, 0.1 mL of an appropriately diluted extract was mixed with 1.9 mL of freshly prepared FRAP reagent. The reaction mixture was incubated for 10 min at 37 °C in the dark, after which the absorbance was measured at 593 nm using a UV–Vis spectrophotometer (6300 Spectrophotometer, Jenway, Stone, UK). The calibration curve was prepared using standard solutions of ferrous sulfate (FeSO_4_) in the concentration range of 0–0.23 mM. The results are expressed as millimoles of Fe^2+^ equivalents per gram of dry weight (mM Fe^2+^/g DW).

### 3.11. ABTS Radical Cation Decolorization Assay

The antioxidant activity of garlic extracts was determined using the ABTS•^+^ decolorization method according to the procedure described by Pavlić et al. [26]. The ABTS radical-cation was generated by reacting a 7 mM ABTS solution with a 2.45 mM potassium persulfate solution in a volume ratio of 1:1 (*v*/*v*). The obtained mixture was left for 16 h in the dark at room temperature for the complete formation of ABTS•^+^ radicals. Immediately before analysis, the working ABTS•^+^ solution was diluted with acetate buffer (pH 3.6) to achieve an initial absorbance of 0.70 ± 0.02 at 734 nm. For the determination of antioxidant activity, 0.1 mL of the appropriately diluted extract was mixed with 2.9 mL of ABTS•^+^ working solution. The reaction mixture was incubated for 300 min at room temperature in the dark, after which the decrease in absorbance was measured at 734 nm using a UV–Vis spectrophotometer (6300 Spectrophotometer, Jenway, Stone, UK). A calibration curve was constructed using freshly prepared Trolox standard solutions in the concentration range 0–0.8 mM. Results are expressed as millimoles of Trolox equivalents per gram of dry weight (mM TE/g DW).

### 3.12. Allicin Determination by LC–MS/MS

Allicin content in garlic extracts was determined using a previously developed and validated liquid chromatography–tandem mass spectrometry (LC–MS/MS) method reported by Krstić et al. [12]. Before analysis, samples were centrifuged at 3000 rpm for 5 min using a centrifuge (MPW-56, MPW Med. Instruments, Warsaw, Poland) to remove suspended particles. The obtained supernatants were filtered through a 0.45 µm membrane filter and transferred into glass vials for LC analysis.

Chromatographic separation was performed using an end-capped C18 Aq HPLC column (3 µm, 2.1 × 100 mm; United Chemical Technologies, Bristol, PA, USA) equipped with a C18 Aq pre-column. The LC system consisted of a Shimadzu LC-20ADXR pump, SIL-20ACXR autosampler, and CTO-20AC column oven. Separation was carried out using gradient elution with a binary mobile phase consisting of acetonitrile (A) and water acidified with 0.1% (*v*/*v*) formic acid (B). The mobile phase flow rate was maintained at 0.25 mL/min. Mass spectrometric detection was performed using a Triple Quad 6500+ LC–MS/MS system (SCIEX, Framingham, MA, USA) operated in positive electrospray ionization mode (ESI+) using multiple reaction monitoring (MRM) mode. The monitored transitions for allicin determination were *m*/*z* 163 → 73, 163 → 87, and 163 → 121, with collision energies of 17, 10, and 10 V, respectively. Allicin detection was performed by combining UV and tandem mass spectrometry detection. UV detection was carried out at 240 nm, while MS/MS detection was used to improve the selectivity and sensitivity of allicin determination. Quantification of allicin was performed by external calibration using freshly prepared allicin standard solutions. The analytical validation parameters, including specificity, linearity, precision, accuracy, limit of detection (LOD), and limit of quantification (LOQ), were previously established and reported by Krstić et al. [12]. The use of the same validated quantitative analytical approach enabled direct comparison of allicin recovery between the extraction technologies investigated in the present study and those previously evaluated by Krstić et al. [12].

### 3.13. Experimental Design and Statistical Analysis

Experimental design and statistical analysis of data obtained during MAE and UAE optimization were carried out using RSM. For both extraction procedures, CCD was used to examine the influence of selected independent process variables and develop quadratic regression models.

The relationship between the independent variables and response variables was described using a second-order polynomial equation:

(1) $$Y\;=\;\beta_{0}\;+\;\sum_{i=1}^{4}\beta_{i}X_{i}+\sum_{i=1}^{4}\beta_{\mathrm{ii}}X_{i}^{2}+\sum \sum_{i<j=1}^{4}\beta_{\mathrm{ij}}X_{i}X_{j}$$

where Y represents the predicted response, β_0_ is the intercept coefficient, β_i_ represents the linear coefficients, β_ii_ represents the quadratic coefficients, β_ij_ represents the interaction coefficients, and X_i_ and X_j_ are the coded independent variables [20].

Four independent variables were analyzed for the MAE process: ethanol concentration, extraction time, liquid-solid ratio, and microwave radiation power. The total content of TPC, TFC, antioxidant activity determined by DPPH, FRAP and ABTS methods, as well as allicin content, were considered as model responses. For each response, a full second-order polynomial was fitted to the coded factors, including the intercept, all linear terms, all two-factor interactions, and all quadratic terms. The full quadratic form was selected to represent potential curvature and interactions across the central composite design. No terms were removed on the basis of their individual *p*-values; retaining all lower-order terms also preserved model hierarchy. Model significance, lack of fit, R^2^, adjusted R^2^, and predicted R^2^ were considered when assessing whether each fitted equation was suitable for interpretation and optimization.

In the UAE process, the influence of three independent variables was investigated: extraction temperature, extraction time and ethanol concentration. Model responses included TPC, TFC and antioxidant activity determined by DPPH, FRAP and ABTS methods. Although allicin content was determined for all UAE extracts, because the corresponding quadratic model did not meet the statistical criteria for adequacy, optimization was not performed using this response.

Experimental design, regression analysis, and optimization were conducted using Design-Expert v.12 software (Stat-Ease Inc., Minneapolis, MN, USA). The significance of the model and individual coefficients was assessed by analysis of variance (ANOVA) at the 95% confidence level (*p* < 0.05). Visualization of the model was performed using response surfaces, contour and perturbation diagrams.

Multi-response optimization was performed using the desirability function. The MAE optimization included TPC, TFC, DPPH, FRAP, ABTS, and allicin, whereas the UAE optimization included TPC, TFC, DPPH, FRAP, and ABTS. The process factors were restricted to the experimental ranges shown in Table 7. For each included response, an individual desirability was calculated from its specified optimization goal and lower and upper response limits. The individual desirabilities were then combined to obtain the overall desirability (D). For each experimental condition defined by the CCD, a single extraction was performed and the resulting extract was analyzed in three analytical replicates. The experimental unit was therefore the individual extraction performed under each specified set of process conditions, whereas the repeated analytical measurements were used to assess the analytical repeatability of the corresponding extract. For each experimental condition, the mean value obtained from the three analytical measurements was used as the response value entered into Design-Expert for response surface modeling. Thus, the analytical replicates were not treated as independent observations in the RSM analysis.

At each selected optimum, one extraction was performed. The resulting extract was measured in three analytical replicates, and the mean ± standard deviation of those measurements is reported in Table 3 and Table 6. These replicates characterize analytical repeatability within one extract; they do not estimate variability between independent extractions. Consequently, no statistical confirmation test or prediction-interval comparison was performed at either optimum.

## 4. Conclusions

RSM was used to examine MAE and UAE conditions and to identify exploratory multi-response compromise settings for garlic extraction. Model adequacy differed among responses: MAE TFC and UAE DPPH and ABTS had limited predictive performance, and the MAE allicin model showed significant lack of fit. Because these responses remained in the desirability calculations, the selected conditions should not be regarded as statistically robust optima supported equally by all included models. The single extraction performed at each selected setting provided a descriptive experimental check, not independent replicated confirmation of predictive performance. The obtained results showed that MAE and UAE represent complementary extraction strategies with different extraction profiles for specific groups of bioactive compounds. Under the selected conditions, the MAE extraction step was shorter and yielded a higher measured allicin value, whereas the UAE extract had higher measured TPC and TFC values. These are descriptive differences between the tested settings rather than evidence of statistical superiority of either technology. These results indicate that the choice of extraction technology should be adapted to the specific purpose of the extract and the desired chemical profile of the bioactive components. When compared with previously reported PLE extraction of the same garlic genotype, the investigated MAE and UAE approaches provided competitive extraction performance under less severe operating conditions, highlighting their potential as alternative green extraction strategies. Overall, the obtained results demonstrate that RSM is a useful tool for optimizing garlic extraction processes and tailoring extract composition according to the targeted bioactive profile and intended application. The findings should be interpreted within the investigated experimental range and for the garlic genotype studied, while further studies with other genotypes and experimental conditions would be useful to confirm the broader applicability of the optimized conditions.

## Figures and Tables

**Table 1:** Central composite design matrix with experimental values of TPC, TFC, antioxidant activity and allicin content for MAE optimization.

Run	Ethanol Concentration (%)	Extraction Time (min)	Liquid-Solid Ratio (mL/g)	Irradiation Power (W)	TPC (g GAE/100 g DW)	TFC (g CE/100 g DW)	DPPH (mM TE/g)	FRAP (mM Fe^2+^/g)	ABTS (mM TE/g)	Allicin (mg/100 g)
1	45	1	30	400	235.02	29.83	5.59	7.53	10.83	49.42
2	75	20	30	400	157.53	44.34	5.49	7.43	10.16	51.77
3	60	4	30	600	254.11	36.28	6.05	9.33	12.67	61.20
4	75	7	10	800	134.11	30.72	2.73	6.60	5.78	19.82
5	75	7	10	400	153.95	31.20	2.36	6.23	5.61	22.24
6	60	4	20	600	259.98	30.63	5.12	10.83	10.39	39.95
7	75	1	10	400	114.64	14.36	2.19	3.72	4.28	23.82
8	45	1	30	800	272.08	38.43	5.80	7.99	11.58	48.13
9	60	1	20	600	221.80	25.85	4.71	7.29	8.59	38.36
10	60	4	20	600	227.04	29.91	4.98	9.94	10.01	39.29
11	75	1	30	800	96.88	26.06	4.75	5.80	7.96	46.05
12	60	4	20	400	218.06	26.09	4.85	8.86	9.51	37.82
13	75	1	10	800	113.52	15.20	2.38	4.54	4.71	22.03
14	45	1	10	400	219.08	26.54	3.08	6.83	5.87	13.13
15	75	1	30	400	105.86	28.75	4.61	5.40	7.02	45.52
16	75	4	30	800	142.93	35.20	5.17	7.09	9.32	54.64
17	75	4	20	600	154.42	21.08	4.36	7.36	8.04	36.73
18	60	4	20	600	247.25	31.35	5.09	9.31	10.11	38.73
19	45	7	30	400	319.25	41.11	6.28	9.82	12.35	58.57
20	60	4	20	600	226.29	28.96	5.54	8.74	9.45	38.43
21	60	4	20	600	227.04	33.02	5.54	8.65	9.84	40.32
22	60	7	20	600	244.26	29.68	4.99	8.78	9.85	40.56
23	60	4	20	800	153.46	19.64	3.83	7.10	7.50	32.50
24	45	1	10	800	278.97	40.63	3.46	7.35	6.64	14.40
25	45	7	10	400	272.24	38.48	3.61	8.26	7.26	15.31
26	45	7	30	800	303.53	37.89	6.09	8.84	11.19	51.18
27	60	4	10	600	189.13	25.35	3.53	8.35	6.78	14.47
28	45	4	20	600	325.11	32.06	5.33	8.00	9.68	34.81
29	45	7	10	800	219.08	25.71	3.32	7.26	7.64	14.09

Abbreviations: MAE, microwave-assisted extraction; TPC, total phenolic content; TFC, total flavonoid content; DPPH, 2,2-diphenyl-1-picrylhydrazyl radical-scavenging assay; FRAP, ferric reducing antioxidant power; ABTS, 2,2′-azinobis(3-ethylbenzothiazoline-6-sulfonic acid) radical-cation assay; GAE, gallic acid equivalents; CE, catechin equivalents; TE, Trolox equivalents; DW, dry weight; Fe^2+^, ferrous ion equivalents.

**Table 2:** Statistical parameters of the developed RSM models for MAE optimization.

Response Variable	R^2^	Adjusted R^2^	Predicted R^2^	*p*-Value of the Model	Lack-of-Fit (*p*-Value)	Significant Process Factors (*p*-Value)
TPC	0.9443	0.8885	0.7066	<0.0001	0.2027	Ethanol concentration (<0.0001); extraction time (0.0076)
TFC	0.8326	0.6653	−0.0270	0.0025	0.0195	Ethanol concentration (0.0031); extraction time (0.0018); liquid-solid ratio (0.0016)
DPPH	0.9694	0.9387	0.8869	<0.0001	0.3961	Ethanol concentration (<0.0001); extraction time (0.0163); liquid-solid ratio (<0.0001)
FRAP	0.8781	0.7563	0.6084	0.0003	0.7300	Ethanol concentration (0.0001); extraction time (0.0010); liquid-solid ratio (0.0093)
ABTS	0.9552	0.9104	0.7814	<0.0001	0.0716	Ethanol concentration (<0.0001); extraction time (0.0010); liquid-solid ratio (<0.0001)
Allicin	0.9765	0.9530	0.8775	<0.0001	0.0050	Liquid-solid ratio (<0.0001)

Abbreviations: RSM, response surface methodology; TPC, total phenolic content; TFC, total flavonoid content; DPPH, 2,2-diphenyl-1-picrylhydrazyl radical-scavenging assay; FRAP, ferric reducing antioxidant power; ABTS, 2,2′-azinobis(3-ethylbenzothiazoline-6-sulfonic acid) radical-cation assay; R^2^, coefficient of determination; *p*, probability value for the indicated statistical test. Adjusted R^2^ accounts for the number of model terms; predicted R^2^ describes predictive performance.

**Table 3:** Optimal conditions and predicted response values for the MAE process.

Input and Output Parameters	Optimal Conditions	
	Predicted Values	Experimental Values
Ethanol concentration (%)	49.3	
Extraction time (min)	7	
Liquid-solid ratio (mL/g)	30	
Microwave power (W)	533	
TPC (g GAE/100 g)	325.12	321.80 ± 5.40
TFC (g CE/100 g)	42.41	40.21 ± 1.20
DPPH (mM TE/g)	6.68	6.52 ± 0.18
FRAP (mM Fe^2+^/g)	10.06	9.84 ± 0.31
ABTS (mM TE/g)	13.03	12.76 ± 0.42
Allicin (mg/100 g)	60.09	58.29 ± 2.10

Abbreviations: TPC, total phenolic content; TFC, total flavonoid content; DPPH, 2,2-diphenyl-1-picrylhydrazyl radical-scavenging assay; FRAP, ferric reducing antioxidant power; ABTS, 2,2′-azinobis(3-ethylbenzothiazoline-6-sulfonic acid) radical-cation assay; GAE, gallic acid equivalents; TE, Trolox equivalents; Fe^2+^, ferrous ion equivalents; SD, standard deviation. Experimental values are the mean ± SD of three analytical measurements of one extract obtained from one extraction at the selected conditions. The single extraction performed at the selected MAE conditions provided an experimental check of the model predictions (Table 3). The measured values were numerically close to the predicted values for most responses. Because the reported standard deviations arise from repeated analyses of one extract, this comparison does not provide independent replicated confirmation of predictive performance. In particular, agreement at this one point does not resolve the significant lack of fit of the TFC and allicin models.

**Table 4:** Central composite design matrix with experimental values of TPC, TFC, antioxidant activity and allicin content for UAE optimization.

Run	Temperature (°C)	Extraction Time (min)	Ethanol Concentration (%)	TPC (g GAE/100 g DW)	TFC (g CE/100 g DW)	DPPH (mM TE/g)	FRAP (mM Fe^2+^/g)	ABTS (mM TE/g)	Allicin (mg/100 g)
1	50	20	45	309.39	97.75	2.04	14.64	7.12	28.43
2	40	40	60	298.91	84.61	1.87	13.71	7.35	32.22
3	50	60	75	126.72	41.38	0.96	6.19	7.15	27.75
4	30	20	45	341.59	102.05	2.17	14.33	6.82	24.19
5	40	20	60	273.46	69.32	1.69	12.19	7.19	31.64
6	50	60	45	313.88	100.85	1.59	14.09	6.99	24.87
7	50	40	60	303.40	77.68	2.29	14.36	7.78	30.08
8	40	40	75	116.24	35.89	1.22	6.19	7.06	31.37
9	30	60	75	126.72	28.48	1.12	5.79	6.64	30.31
10	40	40	45	370.03	121.63	1.92	14.06	6.62	35.02
11	50	20	75	102.01	26.57	0.99	4.90	5.98	27.97
12	30	40	60	285.44	77.68	1.92	12.23	7.05	30.03
13	40	40	60	248.75	76.25	1.88	11.44	7.44	32.14
14	40	40	60	267.47	55.23	1.96	12.88	7.48	33.28
15	40	40	60	277.20	75.77	1.74	12.22	7.45	32.47
16	40	40	60	262.23	72.19	1.88	12.92	7.37	32.64
17	40	60	60	283.19	85.57	1.92	13.22	7.32	32.36
18	40	40	60	271.96	81.03	1.90	13.27	7.31	32.35
19	30	20	75	83.30	19.40	0.93	5.26	5.90	34.73
20	30	60	45	364.79	131.90	2.56	16.97	7.22	29.36

Abbreviations: UAE, ultrasound-assisted ex-traction; TPC, total phenolic content; TFC, total flavonoid content; DPPH, 2,2-diphenyl-1-picrylhydrazyl radical-scavenging assay; FRAP, ferric reducing antioxidant power; ABTS, 2,2′-azinobis(3-ethylbenzothiazoline-6-sulfonic acid) radical-cation assay; GAE, gallic acid equivalents; CE, catechin equivalents; TE, Trolox equivalents; DW, dry weight; Fe^2+^, ferrous ion equivalents.

**Table 5:** Statistical parameters of developed RSM models for UAE optimization of bioactive compounds from garlic.

Response Variable	R^2^	Adjusted R^2^	Predicted R^2^	*p*-Value of the Model	Lack-of-Fit (*p*-Value)	Significant Process Factors (*p*-Value)
TPC	0.9766	0.9555	0.9069	<0.0001	0.3270	Ethanol concentration (<0.0001)
TFC	0.9556	0.9157	0.7540	<0.0001	0.7439	Ethanol concentration (<0.0001); extraction time (0.0278)
DPPH	0.9127	0.8342	0.1854	0.0003	0.0080	Ethanol concentration (<0.0001)
FRAP	0.9637	0.9310	0.6815	<0.0001	0.2526	Ethanol concentration (<0.0001)
ABTS	0.8164	0.6512	−0.8015	0.0100	0.0007	Ethanol concentration (<0.0001); extraction time (0.0278)

Abbreviations: RSM, response surface methodology; TPC, total phenolic content; TFC, total flavonoid content; DPPH, 2,2-diphenyl-1-picrylhydrazyl radical-scavenging assay; FRAP, ferric reducing antioxidant power; ABTS, 2,2′-azinobis(3-ethylbenzothiazoline-6-sulfonic acid) radical-cation assay; R^2^, coefficient of determination; *p*, probability value for the indicated statistical test. Adjusted R^2^ accounts for the number of model terms; predicted R^2^ describes predictive performance.

**Table 6:** Optimal conditions and predicted response values for the UAE process.

Input and Output Parameters	Optimal Conditions	
	Predicted Values	Experimental Values
Extraction temperature (°C)	30	
Extraction time (min)	52.8	
Ethanol concentration (%)	47.5	
TPC (g GAE/100 g DW)	363.12	358.7 ± 6.2
TFC (g CE/100 g DW)	119.57	117.8 ± 3.5
DPPH (mM TE/g)	2.45	2.39 ± 0.09
FRAP (mM Fe^2+^/g)	15.89	15.42 ± 0.39
ABTS (mM TE/g)	7.11	6.82 ± 0.19
Alicin (mg/100 g)	29.00	27.52 ± 0.85

Abbreviations: TPC, total phenolic content; TFC, total flavonoid content; DPPH, 2,2-diphenyl-1-picrylhydrazyl radical-scavenging assay; FRAP, ferric reducing antioxidant power; ABTS, 2,2′-azinobis(3-ethylbenzothiazoline-6-sulfonic acid) radical-cation assay; GAE, gallic acid equivalents; TE, Trolox equivalents; DW, dry weight; Fe^2+^, ferrous ion equivalents; SD, standard deviation. Experimental values are the mean ± SD of three analytical measurements of one extract obtained from one extraction at the selected conditions.

**Table 7:** Coded and actual levels of independent variables used in CCD design for MAE and UAE.

Factor	Independent Variable	Unit	−1 (Low)	0 (Center)	+1 (High)
Microwave-assisted extraction					
A	Ethanol concentration	% (*v*/*v*)	45	60	75
B	Extraction time	min	1	4	7
C	Liquid-to-solid ratio	mL/g	10	20	30
D	Microwave power	W	400	600	800
Ultrasound-assisted extraction					
A	Temperature	°C	30	40	50
B	Extraction time	min	20	40	60
C	Ethanol concentration	% (*v*/*v*)	45	60	75

Abbreviations: CCD, central composite design; MAE, microwave-assisted extraction; UAE, ultrasound-assisted extraction; *v*/*v*, volume per volume. The coded factor levels −1, 0, and +1 correspond to the low, center, and high settings, respectively.

## Data Availability

The data that support the findings of this study are available from the corresponding author upon reasonable request.

## Abbreviations

The following abbreviations are used in this manuscript:

PLE	Pressurized liquid extraction
SWE	Subcritical water extraction
MAE	Microwave-assisted extraction
UAE	Ultrasound-assisted extraction
RSM	Response surface methodology
CCD	Central composite design
TPC	Total phenolic content
TFC	Total flavonoid content
DPPH	2,2-diphenyl-1-picrylhydrazyl
FRAP	Ferric Reducing Antioxidant Power
LC–MS/MS	Liquid chromatography–tandem mass spectrometry
ESI+	Positive electrospray ionization mode
MRM	Multiple reaction monitoring
LOD	Limit of detection
LOQ	Limit of quantification
